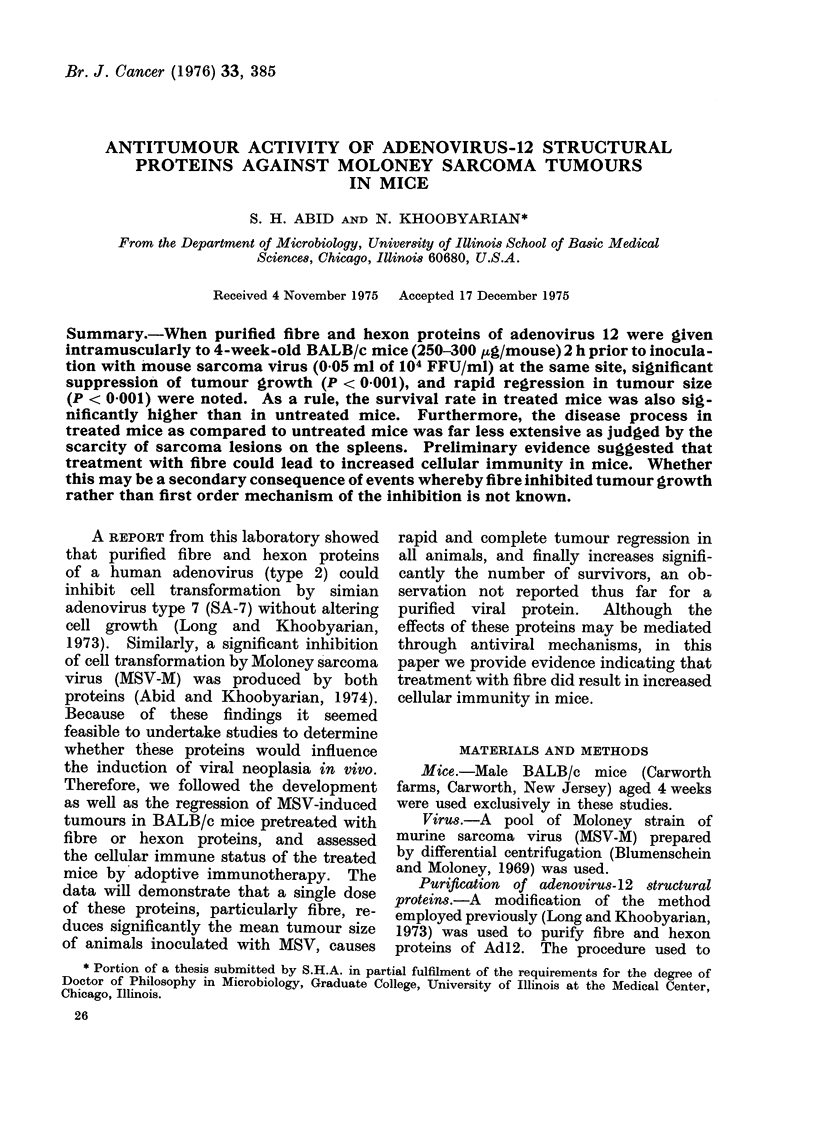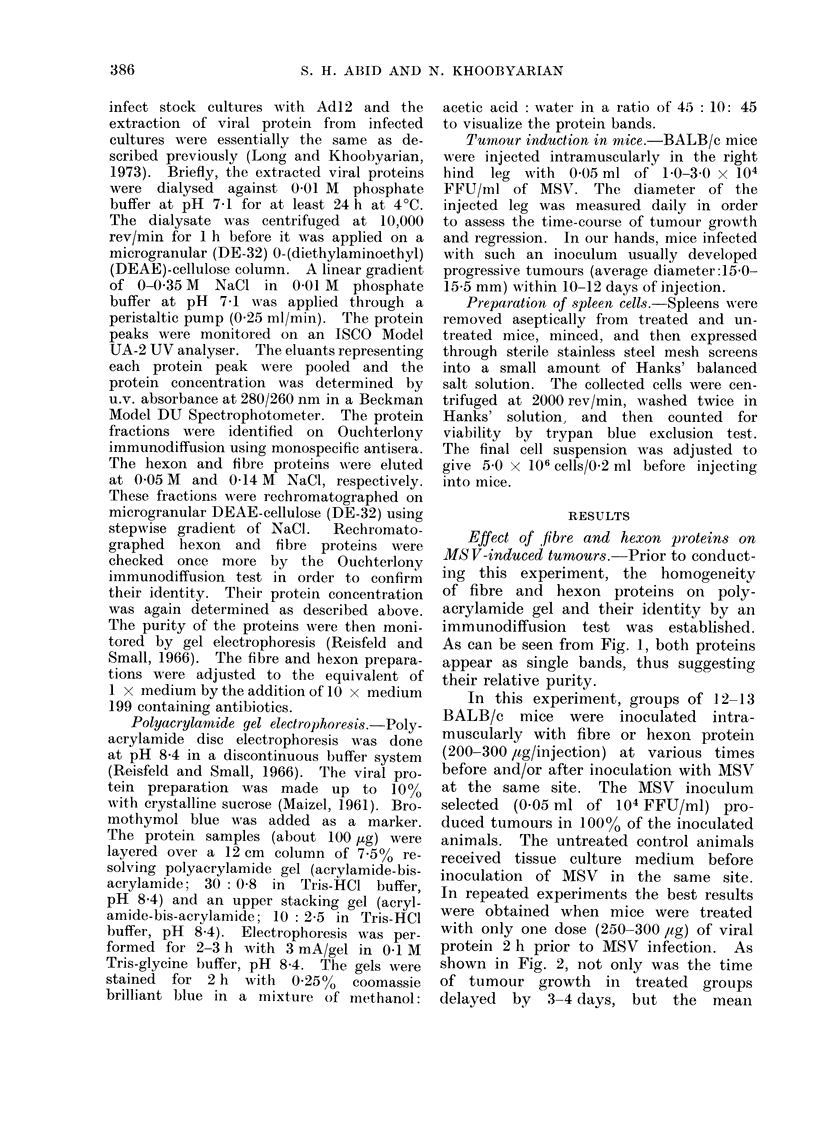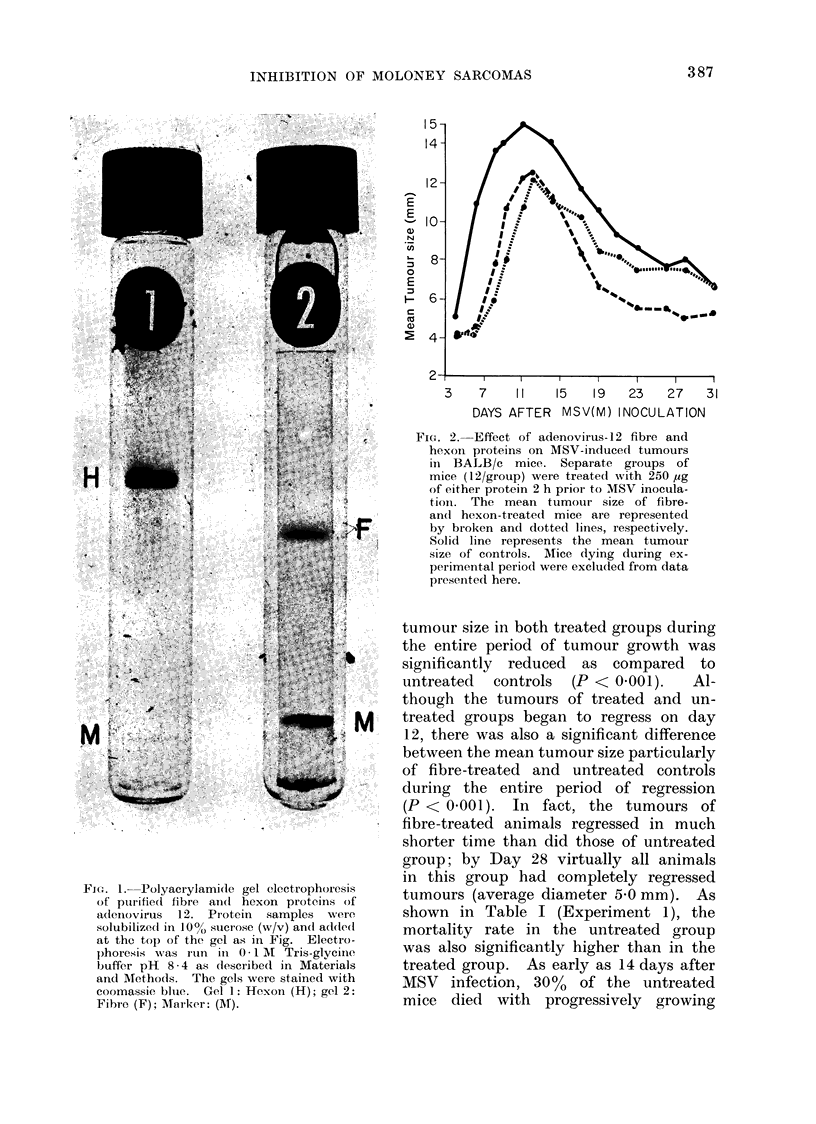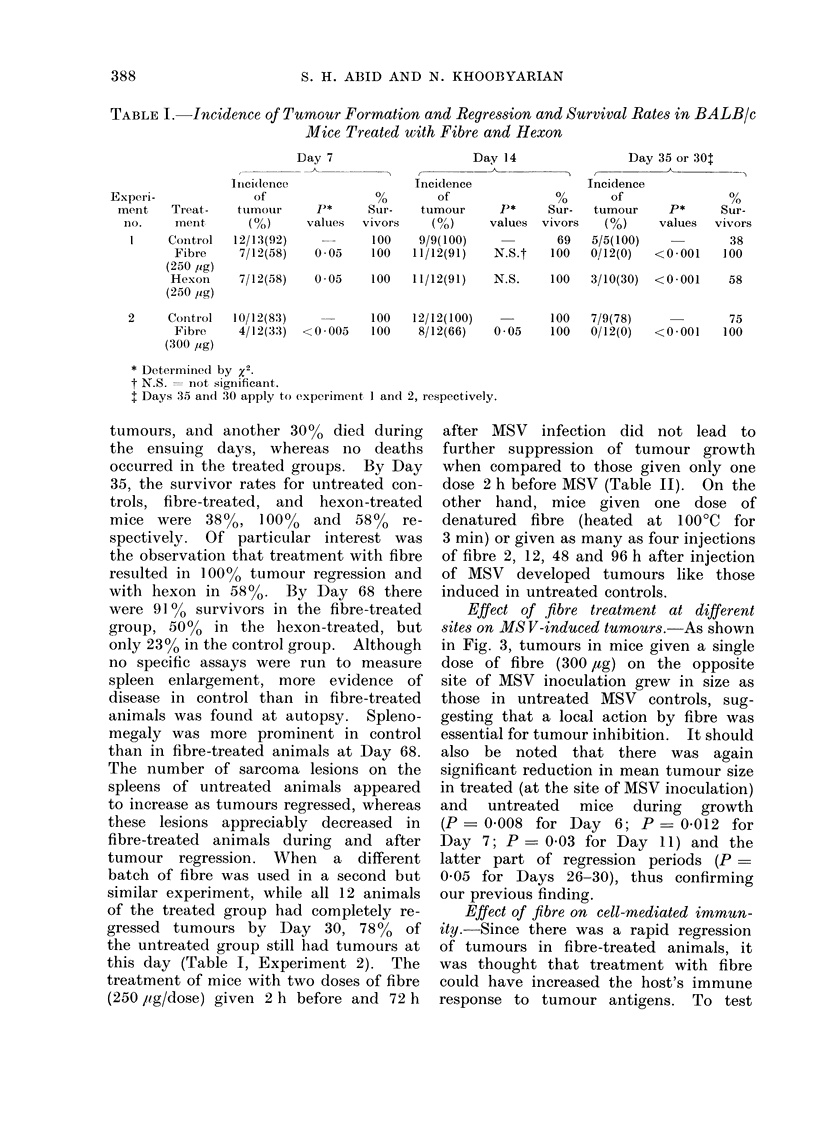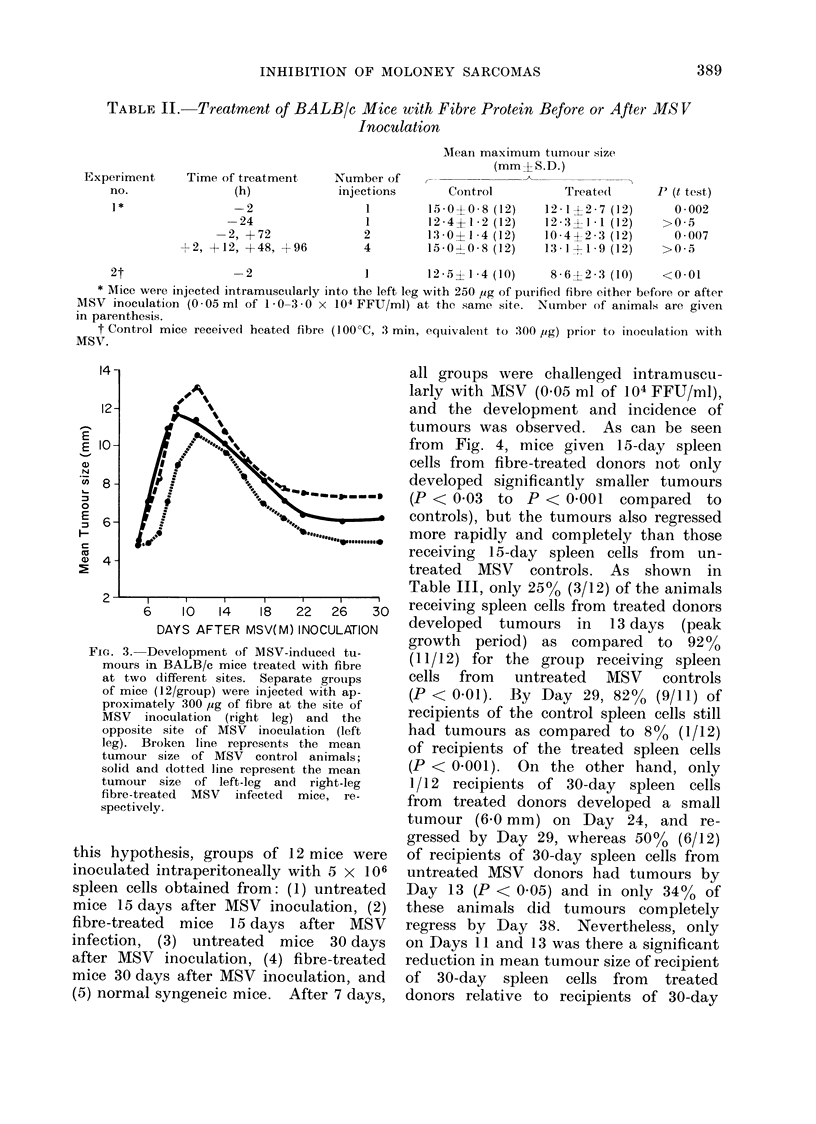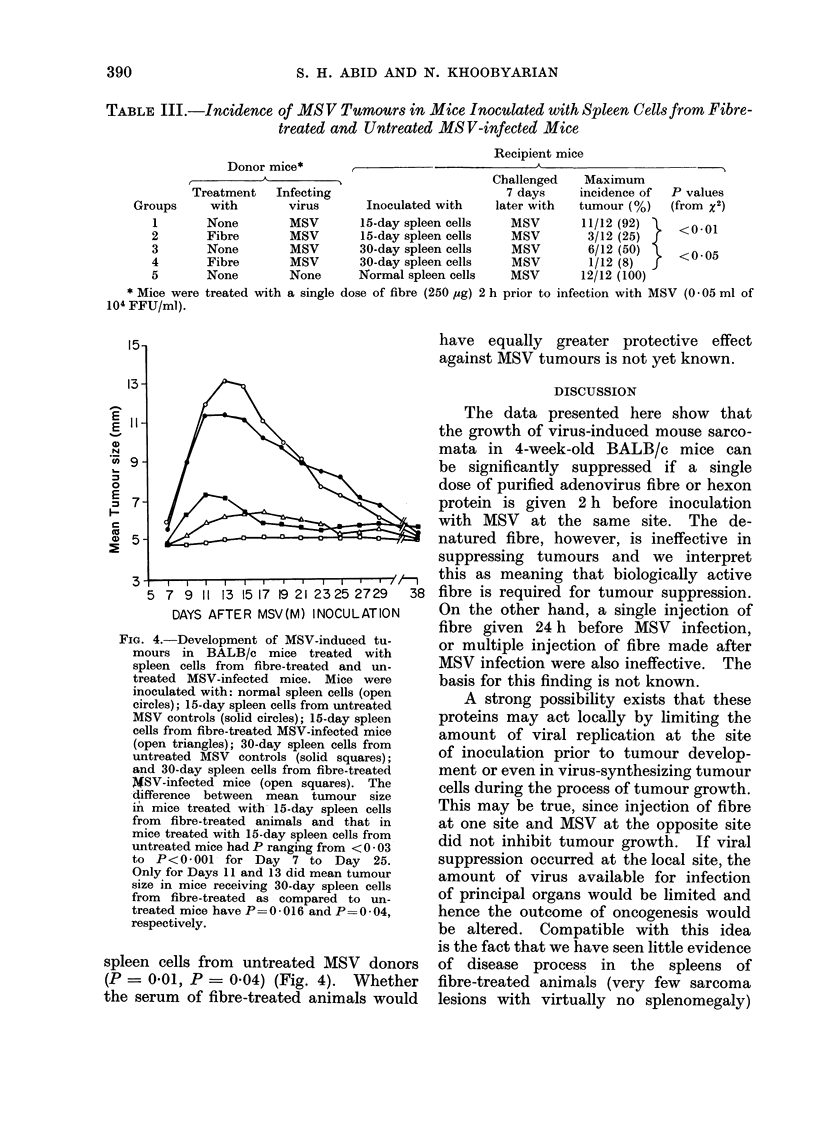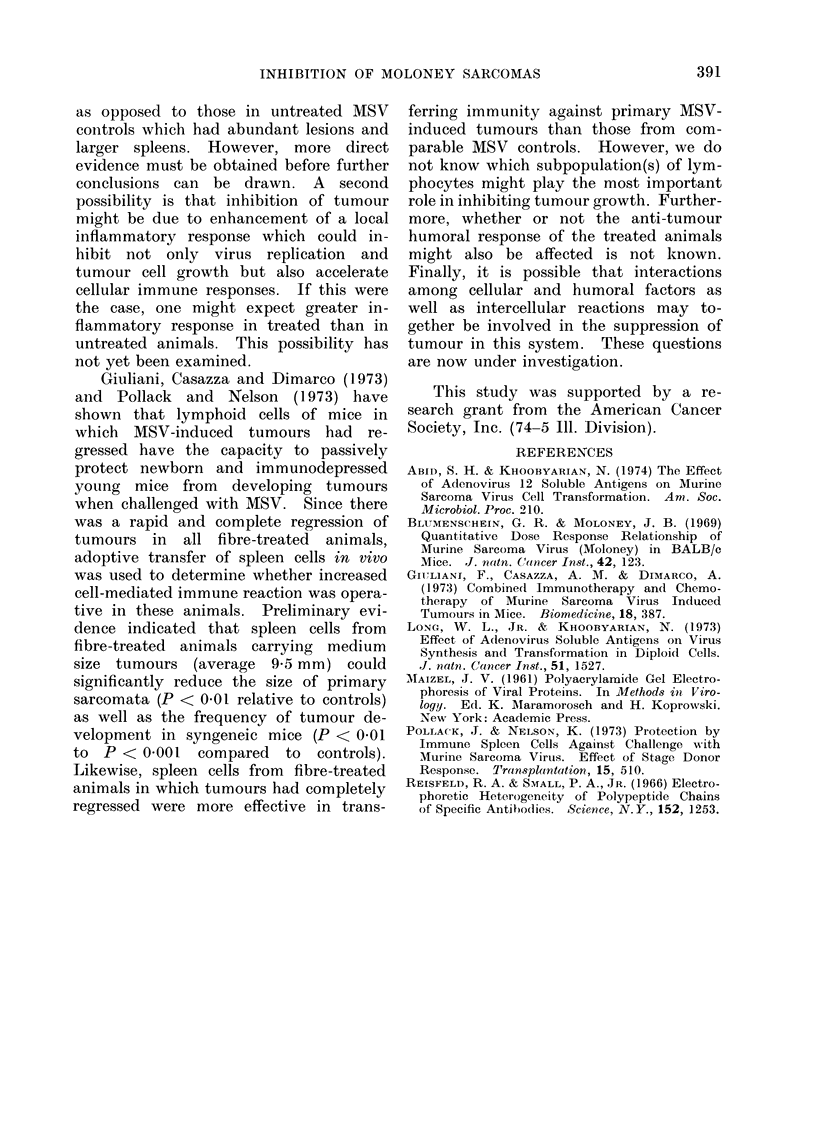# Antitumour activity of adenovirus-12 structural proteins against Moloney sarcoma tumours in mice.

**DOI:** 10.1038/bjc.1976.63

**Published:** 1976-04

**Authors:** S. H. Abid, N. Khoobyarian

## Abstract

**Images:**


Br. J. Cancer (1976) 33, 385

### ANTITUMOUR ACTIVITY OF ADENOVIRUS-12 STRUCTURAL PROTEINS AGAINST MOLONEY SARCOMA TUMOURS IN MICE

S. H. ABID AND N. KHOOBYARIAN*

*From the Department of Microbiology, University of Illinois School of Basic Medical Sciences, Chicago, Illinois 60680, U.S.A.*

Received 4 November 1975 Accepted 17 December 1975

**Summary.—When purified fibre and hexon proteins of adenovirus 12 were given intramuscularly to 4-week-old BALB/c mice (250–300 μg/mouse) 2 h prior to inoculation with mouse sarcoma virus (0·05 ml of $10^4$ FFU/ml) at the same site, significant suppression of tumour growth ($P < 0{\cdot}001$), and rapid regression in tumour size ($P < 0{\cdot}001$) were noted. As a rule, the survival rate in treated mice was also significantly higher than in untreated mice. Furthermore, the disease process in treated mice as compared to untreated mice was far less extensive as judged by the scarcity of sarcoma lesions on the spleens. Preliminary evidence suggested that treatment with fibre could lead to increased cellular immunity in mice. Whether this may be a secondary consequence of events whereby fibre inhibited tumour growth rather than first order mechanism of the inhibition is not known.**

A REPORT from this laboratory showed that purified fibre and hexon proteins of a human adenovirus (type 2) could inhibit cell transformation by simian adenovirus type 7 (SA-7) without altering cell growth (Long and Khoobyarian, 1973). Similarly, a significant inhibition of cell transformation by Moloney sarcoma virus (MSV-M) was produced by both proteins (Abid and Khoobyarian, 1974). Because of these findings it seemed feasible to undertake studies to determine whether these proteins would influence the induction of viral neoplasia *in vivo*. Therefore, we followed the development as well as the regression of MSV-induced tumours in BALB/c mice pretreated with fibre or hexon proteins, and assessed the cellular immune status of the treated mice by adoptive immunotherapy. The data will demonstrate that a single dose of these proteins, particularly fibre, reduces significantly the mean tumour size of animals inoculated with MSV, causes rapid and complete tumour regression in all animals, and finally increases significantly the number of survivors, an observation not reported thus far for a purified viral protein. Although the effects of these proteins may be mediated through antiviral mechanisms, in this paper we provide evidence indicating that treatment with fibre did result in increased cellular immunity in mice.

### MATERIALS AND METHODS

*Mice.*—Male BALB/c mice (Carworth farms, Carworth, New Jersey) aged 4 weeks were used exclusively in these studies.

*Virus.*—A pool of Moloney strain of murine sarcoma virus (MSV-M) prepared by differential centrifugation (Blumenschein and Moloney, 1969) was used.

*Purification of adenovirus-12 structural proteins.*—A modification of the method employed previously (Long and Khoobyarian, 1973) was used to purify fibre and hexon proteins of Ad12. The procedure used to

* Portion of a thesis submitted by S.H.A. in partial fulfilment of the requirements for the degree of Doctor of Philosophy in Microbiology, Graduate College, University of Illinois at the Medical Center, Chicago, Illinois.

infect stock cultures with Ad12 and the extraction of viral protein from infected cultures were essentially the same as described previously (Long and Khoobyarian, 1973). Briefly, the extracted viral proteins were dialysed against 0·01 M phosphate buffer at pH 7·1 for at least 24 h at 4°C. The dialysate was centrifuged at 10,000 rev/min for 1 h before it was applied on a microgranular (DE-32) 0-(diethylaminoethyl) (DEAE)-cellulose column. A linear gradient of 0–0·35 M NaCl in 0·01 M phosphate buffer at pH 7·1 was applied through a peristaltic pump (0·25 ml/min). The protein peaks were monitored on an ISCO Model UA-2 UV analyser. The eluants representing each protein peak were pooled and the protein concentration was determined by u.v. absorbance at 280/260 nm in a Beckman Model DU Spectrophotometer. The protein fractions were identified on Ouchterlony immunodiffusion using monospecific antisera. The hexon and fibre proteins were eluted at 0·05 M and 0·14 M NaCl, respectively. These fractions were rechromatographed on microgranular DEAE-cellulose (DE-32) using stepwise gradient of NaCl. Rechromatographed hexon and fibre proteins were checked once more by the Ouchterlony immunodiffusion test in order to confirm their identity. Their protein concentration was again determined as described above. The purity of the proteins were then monitored by gel electrophoresis (Reisfeld and Small, 1966). The fibre and hexon preparations were adjusted to the equivalent of 1 × medium by the addition of 10 × medium 199 containing antibiotics.

*Polyacrylamide gel electrophoresis.*—Polyacrylamide disc electrophoresis was done at pH 8·4 in a discontinuous buffer system (Reisfeld and Small, 1966). The viral protein preparation was made up to 10% with crystalline sucrose (Maizel, 1961). Bromothymol blue was added as a marker. The protein samples (about 100 μg) were layered over a 12 cm column of 7·5% resolving polyacrylamide gel (acrylamide-bis-acrylamide; 30 : 0·8 in Tris-HCl buffer, pH 8·4) and an upper stacking gel (acrylamide-bis-acrylamide; 10 : 2·5 in Tris-HCl buffer, pH 8·4). Electrophoresis was performed for 2–3 h with 3 mA/gel in 0·1 M Tris-glycine buffer, pH 8·4. The gels were stained for 2 h with 0·25% coomassie brilliant blue in a mixture of methanol: acetic acid : water in a ratio of 45 : 10: 45 to visualize the protein bands.

*Tumour induction in mice.*—BALB/c mice were injected intramuscularly in the right hind leg with 0·05 ml of 1·0–3·0 × $10^4$ FFU/ml of MSV. The diameter of the injected leg was measured daily in order to assess the time-course of tumour growth and regression. In our hands, mice infected with such an inoculum usually developed progressive tumours (average diameter:15·0–15·5 mm) within 10–12 days of injection.

*Preparation of spleen cells.*—Spleens were removed aseptically from treated and untreated mice, minced, and then expressed through sterile stainless steel mesh screens into a small amount of Hanks' balanced salt solution. The collected cells were centrifuged at 2000 rev/min, washed twice in Hanks' solution, and then counted for viability by trypan blue exclusion test. The final cell suspension was adjusted to give 5·0 × $10^6$ cells/0·2 ml before injecting into mice.

### RESULTS

*Effect of fibre and hexon proteins on MSV-induced tumours.*—Prior to conducting this experiment, the homogeneity of fibre and hexon proteins on polyacrylamide gel and their identity by an immunodiffusion test was established. As can be seen from Fig. 1, both proteins appear as single bands, thus suggesting their relative purity.

In this experiment, groups of 12–13 BALB/c mice were inoculated intramuscularly with fibre or hexon protein (200–300 μg/injection) at various times before and/or after inoculation with MSV at the same site. The MSV inoculum selected (0·05 ml of $10^4$ FFU/ml) produced tumours in 100% of the inoculated animals. The untreated control animals received tissue culture medium before inoculation of MSV in the same site. In repeated experiments the best results were obtained when mice were treated with only one dose (250–300 μg) of viral protein 2 h prior to MSV infection. As shown in Fig. 2, not only was the time of tumour growth in treated groups delayed by 3–4 days, but the mean

FIG. 1.—Polyacrylamide gel electrophoresis of purified fibre and hexon proteins of adenovirus 12. Protein samples were solubilized in 10% sucrose (w/v) and added at the top of the gel as in Fig. Electrophoresis was run in 0·1 M Tris-glycine buffer pH 8·4 as described in Materials and Methods. The gels were stained with coomassie blue. Gel 1: Hexon (H); gel 2: Fibre (F); Marker: (M).

FIG. 2.—Effect of adenovirus-12 fibre and hexon proteins on MSV-induced tumours in BALB/c mice. Separate groups of mice (12/group) were treated with 250 $\mu$g of either protein 2 h prior to MSV inoculation. The mean tumour size of fibre- and hexon-treated mice are represented by broken and dotted lines, respectively. Solid line represents the mean tumour size of controls. Mice dying during experimental period were excluded from data presented here.

tumour size in both treated groups during the entire period of tumour growth was significantly reduced as compared to untreated controls ($P < 0{\cdot}001$). Although the tumours of treated and untreated groups began to regress on day 12, there was also a significant difference between the mean tumour size particularly of fibre-treated and untreated controls during the entire period of regression ($P < 0{\cdot}001$). In fact, the tumours of fibre-treated animals regressed in much shorter time than did those of untreated group; by Day 28 virtually all animals in this group had completely regressed tumours (average diameter 5·0 mm). As shown in Table I (Experiment 1), the mortality rate in the untreated group was also significantly higher than in the treated group. As early as 14 days after MSV infection, 30% of the untreated mice died with progressively growing

TABLE I.—*Incidence of Tumour Formation and Regression and Survival Rates in BALB/c Mice Treated with Fibre and Hexon*

| | | Day 7 | | | Day 14 | | | Day 35 or 30‡ | | |
|---|---|---|---|---|---|---|---|---|---|---|
| Experiment no. | Treatment | Incidence of tumour (%) | $P$* values | % Survivors | Incidence of tumour (%) | $P$* values | % Survivors | Incidence of tumour (%) | $P$* values | % Survivors |
| 1 | Control | 12/13(92) | — | 100 | 9/9(100) | — | 69 | 5/5(100) | — | 38 |
| | Fibre (250 μg) | 7/12(58) | 0·05 | 100 | 11/12(91) | N.S.† | 100 | 0/12(0) | <0·001 | 100 |
| | Hexon (250 μg) | 7/12(58) | 0·05 | 100 | 11/12(91) | N.S. | 100 | 3/10(30) | <0·001 | 58 |
| 2 | Control | 10/12(83) | — | 100 | 12/12(100) | — | 100 | 7/9(78) | — | 75 |
| | Fibre (300 μg) | 4/12(33) | <0·005 | 100 | 8/12(66) | 0·05 | 100 | 0/12(0) | <0·001 | 100 |

* Determined by $\chi^2$.

† N.S. = not significant.

‡ Days 35 and 30 apply to experiment 1 and 2, respectively.

tumours, and another 30% died during the ensuing days, whereas no deaths occurred in the treated groups. By Day 35, the survivor rates for untreated controls, fibre-treated, and hexon-treated mice were 38%, 100% and 58% respectively. Of particular interest was the observation that treatment with fibre resulted in 100% tumour regression and with hexon in 58%. By Day 68 there were 91% survivors in the fibre-treated group, 50% in the hexon-treated, but only 23% in the control group. Although no specific assays were run to measure spleen enlargement, more evidence of disease in control than in fibre-treated animals was found at autopsy. Splenomegaly was more prominent in control than in fibre-treated animals at Day 68. The number of sarcoma lesions on the spleens of untreated animals appeared to increase as tumours regressed, whereas these lesions appreciably decreased in fibre-treated animals during and after tumour regression. When a different batch of fibre was used in a second but similar experiment, while all 12 animals of the treated group had completely regressed tumours by Day 30, 78% of the untreated group still had tumours at this day (Table I, Experiment 2). The treatment of mice with two doses of fibre (250 μg/dose) given 2 h before and 72 h after MSV infection did not lead to further suppression of tumour growth when compared to those given only one dose 2 h before MSV (Table II). On the other hand, mice given one dose of denatured fibre (heated at 100°C for 3 min) or given as many as four injections of fibre 2, 12, 48 and 96 h after injection of MSV developed tumours like those induced in untreated controls.

*Effect of fibre treatment at different sites on MSV-induced tumours.*—As shown in Fig. 3, tumours in mice given a single dose of fibre (300 μg) on the opposite site of MSV inoculation grew in size as those in untreated MSV controls, suggesting that a local action by fibre was essential for tumour inhibition. It should also be noted that there was again significant reduction in mean tumour size in treated (at the site of MSV inoculation) and untreated mice during growth ($P = 0{\cdot}008$ for Day 6; $P = 0{\cdot}012$ for Day 7; $P = 0{\cdot}03$ for Day 11) and the latter part of regression periods ($P = 0{\cdot}05$ for Days 26–30), thus confirming our previous finding.

*Effect of fibre on cell-mediated immunity.*—Since there was a rapid regression of tumours in fibre-treated animals, it was thought that treatment with fibre could have increased the host's immune response to tumour antigens. To test

TABLE II.—*Treatment of BALB/c Mice with Fibre Protein Before or After MSV Inoculation*

| Experiment no. | Time of treatment (h) | Number of injections | Mean maximum tumour size (mm ± S.D.) Control | Treated | P (t test) |
|---|---|---|---|---|---|
| 1* | −2 | 1 | 15·0±0·8 (12) | 12·1±2·7 (12) | 0·002 |
| | −24 | 1 | 12·4±1·2 (12) | 12·3±1·1 (12) | >0·5 |
| | −2, +72 | 2 | 13·0±1·4 (12) | 10·4±2·3 (12) | 0·007 |
| | +2, +12, +48, +96 | 4 | 15·0±0·8 (12) | 13·1±1·9 (12) | >0·5 |
| 2† | −2 | 1 | 12·5±1·4 (10) | 8·6±2·3 (10) | <0·01 |

* Mice were injected intramuscularly into the left leg with 250 μg of purified fibre either before or after MSV inoculation (0·05 ml of 1·0–3·0 × $10^4$ FFU/ml) at the same site. Number of animals are given in parenthesis.

† Control mice received heated fibre (100°C, 3 min, equivalent to 300 μg) prior to inoculation with MSV.

FIG. 3.—Development of MSV-induced tumours in BALB/c mice treated with fibre at two different sites. Separate groups of mice (12/group) were injected with approximately 300 μg of fibre at the site of MSV inoculation (right leg) and the opposite site of MSV inoculation (left leg). Broken line represents the mean tumour size of MSV control animals; solid and dotted line represent the mean tumour size of left-leg and right-leg fibre-treated MSV infected mice, respectively.

this hypothesis, groups of 12 mice were inoculated intraperitoneally with 5 × $10^6$ spleen cells obtained from: (1) untreated mice 15 days after MSV inoculation, (2) fibre-treated mice 15 days after MSV infection, (3) untreated mice 30 days after MSV inoculation, (4) fibre-treated mice 30 days after MSV inoculation, and (5) normal syngeneic mice. After 7 days, all groups were challenged intramuscularly with MSV (0·05 ml of $10^4$ FFU/ml), and the development and incidence of tumours was observed. As can be seen from Fig. 4, mice given 15-day spleen cells from fibre-treated donors not only developed significantly smaller tumours ($P < 0·03$ to $P < 0·001$ compared to controls), but the tumours also regressed more rapidly and completely than those receiving 15-day spleen cells from untreated MSV controls. As shown in Table III, only 25% (3/12) of the animals receiving spleen cells from treated donors developed tumours in 13 days (peak growth period) as compared to 92% (11/12) for the group receiving spleen cells from untreated MSV controls ($P < 0·01$). By Day 29, 82% (9/11) of recipients of the control spleen cells still had tumours as compared to 8% (1/12) of recipients of the treated spleen cells ($P < 0·001$). On the other hand, only 1/12 recipients of 30-day spleen cells from treated donors developed a small tumour (6·0 mm) on Day 24, and regressed by Day 29, whereas 50% (6/12) of recipients of 30-day spleen cells from untreated MSV donors had tumours by Day 13 ($P < 0·05$) and in only 34% of these animals did tumours completely regress by Day 38. Nevertheless, only on Days 11 and 13 was there a significant reduction in mean tumour size of recipient of 30-day spleen cells from treated donors relative to recipients of 30-day

TABLE III.—*Incidence of MSV Tumours in Mice Inoculated with Spleen Cells from Fibre-treated and Untreated MSV-infected Mice*

| | Donor mice* | | Recipient mice | | | |
|---|---|---|---|---|---|---|
| Groups | Treatment with | Infecting virus | Inoculated with | Challenged 7 days later with | Maximum incidence of tumour (%) | P values (from $\chi^2$) |
| 1 | None | MSV | 15-day spleen cells | MSV | 11/12 (92) | <0·01 |
| 2 | Fibre | MSV | 15-day spleen cells | MSV | 3/12 (25) | |
| 3 | None | MSV | 30-day spleen cells | MSV | 6/12 (50) | <0·05 |
| 4 | Fibre | MSV | 30-day spleen cells | MSV | 1/12 (8) | |
| 5 | None | None | Normal spleen cells | MSV | 12/12 (100) | |

* Mice were treated with a single dose of fibre (250 $\mu$g) 2 h prior to infection with MSV (0·05 ml of $10^4$ FFU/ml).

FIG. 4.—Development of MSV-induced tumours in BALB/c mice treated with spleen cells from fibre-treated and untreated MSV-infected mice. Mice were inoculated with: normal spleen cells (open circles); 15-day spleen cells from untreated MSV controls (solid circles); 15-day spleen cells from fibre-treated MSV-infected mice (open triangles); 30-day spleen cells from untreated MSV controls (solid squares); and 30-day spleen cells from fibre-treated MSV-infected mice (open squares). The difference between mean tumour size in mice treated with 15-day spleen cells from fibre-treated animals and that in mice treated with 15-day spleen cells from untreated mice had $P$ ranging from $<0\cdot03$ to $P<0\cdot001$ for Day 7 to Day 25. Only for Days 11 and 13 did mean tumour size in mice receiving 30-day spleen cells from fibre-treated as compared to untreated mice have $P=0\cdot016$ and $P=0\cdot04$, respectively.

spleen cells from untreated MSV donors ($P$ = 0·01, $P$ = 0·04) (Fig. 4). Whether the serum of fibre-treated animals would have equally greater protective effect against MSV tumours is not yet known.

### DISCUSSION

The data presented here show that the growth of virus-induced mouse sarcomata in 4-week-old BALB/c mice can be significantly suppressed if a single dose of purified adenovirus fibre or hexon protein is given 2 h before inoculation with MSV at the same site. The denatured fibre, however, is ineffective in suppressing tumours and we interpret this as meaning that biologically active fibre is required for tumour suppression. On the other hand, a single injection of fibre given 24 h before MSV infection, or multiple injection of fibre made after MSV infection were also ineffective. The basis for this finding is not known.

A strong possibility exists that these proteins may act locally by limiting the amount of viral replication at the site of inoculation prior to tumour development or even in virus-synthesizing tumour cells during the process of tumour growth. This may be true, since injection of fibre at one site and MSV at the opposite site did not inhibit tumour growth. If viral suppression occurred at the local site, the amount of virus available for infection of principal organs would be limited and hence the outcome of oncogenesis would be altered. Compatible with this idea is the fact that we have seen little evidence of disease process in the spleens of fibre-treated animals (very few sarcoma lesions with virtually no splenomegaly)

as opposed to those in untreated MSV controls which had abundant lesions and larger spleens. However, more direct evidence must be obtained before further conclusions can be drawn. A second possibility is that inhibition of tumour might be due to enhancement of a local inflammatory response which could inhibit not only virus replication and tumour cell growth but also accelerate cellular immune responses. If this were the case, one might expect greater inflammatory response in treated than in untreated animals. This possibility has not yet been examined.

Giuliani, Casazza and Dimarco (1973) and Pollack and Nelson (1973) have shown that lymphoid cells of mice in which MSV-induced tumours had regressed have the capacity to passively protect newborn and immunodepressed young mice from developing tumours when challenged with MSV. Since there was a rapid and complete regression of tumours in all fibre-treated animals, adoptive transfer of spleen cells *in vivo* was used to determine whether increased cell-mediated immune reaction was operative in these animals. Preliminary evidence indicated that spleen cells from fibre-treated animals carrying medium size tumours (average 9·5 mm) could significantly reduce the size of primary sarcomata ($P < 0{\cdot}01$ relative to controls) as well as the frequency of tumour development in syngeneic mice ($P < 0{\cdot}01$ to $P < 0{\cdot}001$ compared to controls). Likewise, spleen cells from fibre-treated animals in which tumours had completely regressed were more effective in transferring immunity against primary MSV-induced tumours than those from comparable MSV controls. However, we do not know which subpopulation(s) of lymphocytes might play the most important role in inhibiting tumour growth. Furthermore, whether or not the anti-tumour humoral response of the treated animals might also be affected is not known. Finally, it is possible that interactions among cellular and humoral factors as well as intercellular reactions may together be involved in the suppression of tumour in this system. These questions are now under investigation.

This study was supported by a research grant from the American Cancer Society, Inc. (74–5 Ill. Division).

REFERENCES

Abid, S. H. & Khoobyarian, N. (1974) The Effect of Adenovirus 12 Soluble Antigens on Murine Sarcoma Virus Cell Transformation. *Am. Soc. Microbiol. Proc.* 210.

Blumenschein, G. R. & Moloney, J. B. (1969) Quantitative Dose Response Relationship of Murine Sarcoma Virus (Moloney) in BALB/c Mice. *J. natn. Cancer Inst.*, **42**, 123.

Giuliani, F., Casazza, A. M. & Dimarco, A. (1973) Combined Immunotherapy and Chemotherapy of Murine Sarcoma Virus Induced Tumours in Mice. *Biomedicine*, **18**, 387.

Long, W. L., Jr. & Khoobyarian, N. (1973) Effect of Adenovirus Soluble Antigens on Virus Synthesis and Transformation in Diploid Cells. *J. natn. Cancer Inst.*, **51**, 1527.

Maizel, J. V. (1961) Polyacrylamide Gel Electrophoresis of Viral Proteins. In *Methods in Virology*. Ed. K. Maramorosch and H. Koprowski. New York: Academic Press.

Pollack, J. & Nelson, K. (1973) Protection by Immune Spleen Cells Against Challenge with Murine Sarcoma Virus. Effect of Stage Donor Response. *Transplantation*, **15**, 510.

Reisfeld, R. A. & Small, P. A., Jr. (1966) Electrophoretic Heterogeneity of Polypeptide Chains of Specific Antibodies. *Science, N.Y.*, **152**, 1253.